# Addison’s disease due to adrenal insufficiency in an immunocompetent patient with disseminated cryptococcosis. Case report

**DOI:** 10.17843/rpmesp.2025.422.14304

**Published:** 2025-06-09

**Authors:** José Paz-Ibarra, Marcio Concepción-Zavaleta, Miguel Tipiani Mallma, Héctor Delgado Nicolás, Hilder Herrera-Silvestre, Laurie Marcilla-Truyenque, Joseph Arzapalo-Benavides, Luis Concepción-Urteaga

**Affiliations:** 1 School of Medicine, National University of San Marcos, Lima, Peru. National University of San Marcos School of Medicine National University of San Marcos Lima Peru; 2 Edgardo Rebagliati Martins National Hospital, Lima, Peru. Edgardo Rebagliati Martins National Hospital Lima Peru; 3 Universidad Científica del Sur, Lima, Peru. Universidad Científica del Sur Universidad Científica del Sur Lima Peru; 4 School of Medicine, National University of Trujillo, Trujillo, Peru. National University of Trujillo School of Medicine National University of Trujillo Trujillo Peru

**Keywords:** Adrenal insufficiency, Addison disease, Cryptococcus

## Abstract

Systemic mycoses, such as cryptococcosis, mainly affect the lungs and central nervous system; however, involvement of other organs, such as the adrenal glands, is rare. This has been described in some cases of primary adrenal insufficiency (PAI) of fungal origin, which are associated with high mortality. We present the case of a 65-year-old immunocompetent man who presented clinical manifestations of adrenal insufficiency. Biochemical tests confirmed PAI, and abdominal tomography revealed hyperplasia of both adrenal glands, predominantly on the left side. Left adrenalectomy was performed, and pathological examination identified granulomas and fungal structures compatible with *Cryptococcus* spp. The patient received hormone replacement therapy and antifungal treatment, with favorable outcome. Cryptococcal adrenalitis should be considered in the differential diagnosis of PAI, particularly in the presence of relevant epidemiological history, and timely treatment is key to improving prognosis.

## INTRODUCTION

Adrenal insufficiency is a clinical disorder characterized by the inability of the adrenal glands to produce adequate amounts of steroid hormones, primarily cortisol and aldosterone. It is classified as primary, secondary, or tertiary adrenal insufficiency, depending on the origin of the dysfunction. Primary adrenal insufficiency (PAI) is caused by the destruction of the adrenal glands ^1^.

The etiologies of primary adrenal insufficiency (PAI) include autoimmune, infectious, neoplastic, and infiltrative causes. Infections are a significant cause of PAI, especially in endemic regions and in immunocompromised patients ^1^. Historically, tuberculosis has been the leading infectious cause of PAI ^2^; however, in the modern era, the prevalence of other infections, such as cryptococcal adrenalitis, has increased due to the expansion of the HIV/AIDS pandemic and other immunosuppression states ^3^.

*Cryptococcus* spp. is an encapsulated fungus that causes cryptococcosis, a systemic infection that predominantly affects individuals with severe immunodeficiency. The infection is acquired through inhalation of spores from the environment, allowing hematogenous dissemination to various organs, including the central nervous system and adrenal glands ^4^. It mainly affects immunocompromised individuals, although it can also occur in immunocompetent hosts ^5^.

The clinical manifestations of cryptococcal adrenalitis can be difficult to distinguish from other causes of adrenal insufficiency. Common symptoms include fatigue, anorexia, weight loss, hypotension, skin hyperpigmentation, hypoglycemia, hyponatremia, and hypercalcemia ^6^. However, in cryptococcal adrenalitis, these symptoms may be accompanied by neurological or respiratory manifestations due to systemic dissemination of the infection, further complicating diagnosis ^4^.

The diagnosis of cryptococcal adrenalitis requires a high index of clinical suspicion. Imaging studies, such as computed tomography (CT) or magnetic resonance imaging (MRI) of the adrenal glands, may reveal bilateral adrenal enlargement ^7^. Diagnostic confirmation is usually obtained through fungal cultures, cryptococcal antigen testing in serum or cerebrospinal fluid ^4^, and, in some cases, adrenal biopsy ^6^.

The treatment of choice for systemic cryptococcosis with adrenal involvement includes antifungal therapy with agents such as liposomal amphotericin B and flucytosine ^8^, the latter not available in Peru, along with hormone replacement therapy (glucocorticoids and mineralocorticoids). In countries such as ours, there are recommendations for use and successful experience with treatment based on amphotericin B deoxycholate and fluconazole ^9^^,^^10^.

Early treatment is essential to prevent adrenal crisis. Response to treatment depends on the severity of the infection and the patient’s immune status, therefore, close monitoring is essential ^11^. This report aimed to describe the case of an elderly immunocompetent male patient who developed primary adrenal insufficiency caused by *Cryptococcus* spp.

## CASE REPORT

We present the case of a 65-year-old Peruvian patient, a veterinarian with more than 25 years of experience in livestock management. He was born in the city of Huamanga, Ayacucho, Peru, but has lived in Lima, Peru, for more than 25 years. He was diagnosed with type 2 diabetes mellitus (DM2) 11 years ago and treated with metformin 850 mg. His medical history also includes an ischemic stroke 4 years ago, with no sequelae.

**Figure 1:**
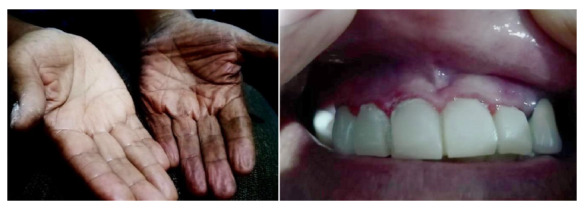
Hyperpigmentation in the flexures of the palm and gingival mucosa.

In December 2023, he was admitted to the Edgardo Rebagliati Martins National Hospital, a national referral hospital, with the following vital signs: blood pressure of 80/60 mm Hg, heart rate of 97 beats per minute, afebrile, and no meningeal signs. He was disoriented and drowsy, with hyperpigmentation of the skin and mucous membranes (Figure 1), and no palpable abdominal masses. Biochemical analyses revealed serum sodium of 113 mmol/L and serum potassium of 5.2 mmol/L. Suspecting an adrenal crisis, serum cortisol was measured and hydration started along with hydrocortisone 100 mg IV every 8 hours. PAI and controlled diabetes were diagnosed with the following results: Basal cortisol: 5.77 ug/dL (R.V: 3.7-19.4); plasma ACTH: 197 pg/mL (R.V: 0-46); DHEA-S: 18.20 ug/dL (R.V.: 33.6-78.9); plasma aldosterone: <10 pg/mL (R.V: 10-160); TSH: 1.12 uUI/mL (R.V: 0.4-4); Free T4: 1.6 ng/dL (R.V: 0.8-1.9); Antithyroid antibodies: (-); HbA1C: 6.1%; and therapy started with prednisone 7.5 mg/day and fludrocortisone 0.1 mg/day, achieving hemodynamic and biochemical improvement.

**Figure 2:**
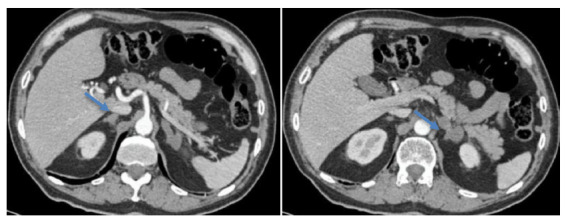
Abdominal cross-sectional tomography showing bilateral nodular adrenal hyperplasia with predominance on the left side (blue arrows).

**Figure 3:**
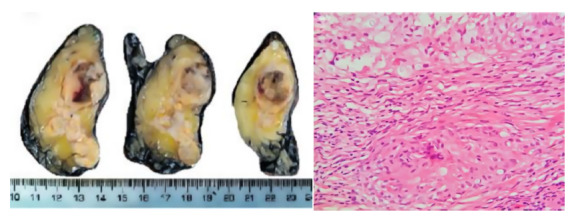
In the macroscopic image, the left adrenal gland shows brownish-yellow areas with a necrotic center and friable consistency (a). The microscopic image shows an extensive chronic granulomatous inflammatory process with necrosis, hemorrhage, and multinucleated giant cells surrounding translucent rounded and oval structures (H&E, 40x) (b).

**Figure 4:**
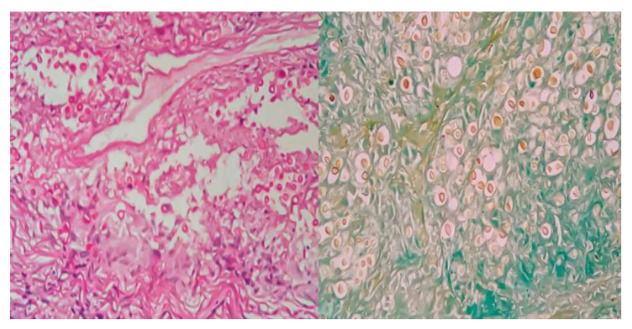
Microscopic image at 40x magnification, the rounded fungal structures were stained using histochemical techniques with PAS staining (a) and Grocott staining (b), respectively.

As part of the etiological study, sputum and gastric lavage tests were performed for acid-alcohol resistant bacilli, pancultures, and tuberculin tests, all with negative results; a cryptolatex test on serum was positive. TORCH serology (toxoplasmosis, rubella, cytomegalovirus, and herpes simplex virus), as well as serology for hepatitis B and C, HTLV 1-2, and HIV, were negative. Antinuclear antibodies, anti-thyroid peroxidase, and antithyroglobulin were also negative. The chest X-ray was normal, and computed tomography (CT) of the adrenal glands showed bilateral enlargement, with a thickness of 11 mm in the right adrenal gland and 14 mm in the left, both with a hypodense nodular appearance and non-contrast attenuation values of 45 HU and 25 HU for the right and left adrenal glands, respectively (Figure 2). respectively (Figure 2). A medical multidisciplinary meeting recommended left adrenalectomy. Pathological examination revealed granulomatous inflammation with hemorrhagic necrosis and rounded fungal structures consistent with *Cryptococcus* spp. (Figures 3 and 4). The diagnosis of cryptococcal adrenalitis caused by *Cryptococcus* spp. was confirmed.

A lumbar puncture was performed due to persistent holocranial headache, predominantly in the occipital region. An opening pressure of 15 cm of water was obtained, Indian ink staining was negative, latex agglutination for *Cryptococcus* was positive (quantitative test 1:256), and CSF culture was negative. It was concluded that the patient had central nervous system (CNS) involvement. The final diagnosis was systemic cryptococcosis with CNS and adrenal gland involvement. The patient was treated with fluconazole 400 mg/day and amphotericin B deoxycholate 1 mg/kg/day intravenously (intensive phase) for 2 weeks, followed by an additional 4 weeks of amphotericin B. The patient showed favorable progress and was discharged with a diagnosis of primary adrenal insufficiency (PAI), with outpatient treatment of oral fluconazole for 6 months (consolidation phase), prednisone 5 mg once daily, and fludrocortisone 0.1 mg once daily, which he continues to take. No signs of renal, hepatic, or cardiovascular toxicity associated with the use of amphotericin B and fluconazole were identified.

## DISCUSSION

Autoimmune adrenalitis is the main cause of primary adrenal insufficiency (PAI), occurring mainly between the ages of 30 and 50 and in females ^12^^,^^13^. The reported case involved a 65-year-old male patient, which led us to consider other causes of PAI. Adrenal CT, which showed bilateral enlargement of the adrenal glands, along with the patient’s history as a veterinarian working with livestock, led us to perform an adrenal biopsy to identify the etiology.

Cryptococcosis is an infectious disease caused by two agents: *Cryptococcus neoformans* and *Cryptococcus gattii*, which can affect the lungs, meninges, skin, central nervous system, and adrenal glands ^14^. It is traditionally considered an opportunistic infection in immunocompromised patients ^14^. However, in recent years, there has been an increase in the incidence of this disease in immunocompetent individuals and those with mild to moderate immunodeficiency. In these cases, the infection may be asymptomatic and resolve completely without antifungal treatment, although, in rare cases, it can progress to a serious disease with dissemination and development of cryptococcal meningitis, as happened in our case ^14^^,^^15^.

The clinical manifestations of *Cryptococcus* spp. infection are nonspecific and can range from an insidious onset to a severe presentation. Central nervous system infection may manifest with headache, nausea and vomiting, general malaise, altered mental status, fever, and seizures ^16^. An atypical and very rare presentation of *Cryptococcus* spp. infection is cryptococcal meningitis, which occurs when 90% or more of the adrenal cortex has been destroyed ^17^, reaching the adrenal glands via the bloodstream due to their extensive vascularization ^18^^,^^19^.

The hypothesis is that *Cryptococcus* spp. can affect the adrenal glands in two ways. The first is directly, in which *Cryptococcus neoformans* colonizes and proliferates in the adrenal tissue. The damage is caused by fungal invasion and the resulting inflammatory response. The fungal capsule, composed mainly of polysaccharides such as glucuronoxylomannan, facilitates adhesion to adrenocortical cells and resistance to phagocytosis. This invasion leads to chronic inflammation and granuloma formation in the adrenal tissue, causing adrenalitis. Local inflammation causes necrosis of the adrenocortical tissue, leading to adrenal insufficiency ^18^^,^^20^. The second is an indirect way, in which the presence of an infectious agent elsewhere in the body activates the hypothalamic-pituitary-adrenal axis. This prolonged activation leads to hypercortisolism, which could affect immune function, especially T cell-mediated immunity ^18^.

There is no pathognomonic sign for cryptococcal adrenalitis. The clinical presentation corresponds to that of an PAI and/or adrenal crisis. Compared to the few published cases, the main reported clinical manifestations include fatigue, nausea and vomiting, anorexia, abdominal pain, diarrhea, hypotension, hyponatremia, and hypokalemia. It is important to note that no cases of isolated cryptococcal adrenalitis have been documented; it has always been part of dissemination from a primary focus, usually meningeal and/or pulmonary ^21^^,^^22^^,^^23^.

Few prospective studies offer specific guidelines for the treatment of cryptococcosis in patients without HIV and without transplantation, so the same guidelines are used for patients with HIV. Although current management guidelines recommend the use of agents such as liposomal amphotericin B and flucytosine as first-line therapy ^8^, the use of amphotericin B combined with fluconazole, followed by a consolidation phase with fluconazole for 6 to 12 months, has shown good results in clinical practice. This was evidenced by the favorable outcome of our patient with the antifungal treatment initiated ^24^^,^^25^.

The prognosis for adrenal function in patients with cryptococcal adrenalitis receiving antifungal treatment varies and depends on the degree of adrenal damage and response to treatment. Although amphotericin B and fluconazole can control the infection, adrenal damage is usually irreversible, resulting in permanent adrenal insufficiency and the need for long-term hormone replacement therapy ^18^^,^^20^.

In conclusion, cryptococcal adrenalitis is a very rare, nonspecific condition that is difficult to diagnose in patients without detectable immunosuppression. However, it is important to consider it in the differential diagnosis of PIA. In our case, timely initiation of antifungal treatment allowed for a favorable clinical outcome for the patient.
