# Supplementary material for: Addison’s disease due to adrenal insufficiency in an immunocompetent patient with disseminated cryptococcosis. Case report
Source: Rev Peru Med Exp Salud Publica. 2025 Jun 9;42(2):217–22. doi: 10.17843/rpmesp.2025.422.14304 (PMC12377890; doi:10.17843/rpmesp.2025.422.14304)
Supplement: Translation

# Translation: Enfermedad de Addison por afectación suprarrenal en un paciente inmunocompetente con criptococosis diseminada: reporte de caso

**Authors:** José Paz-Ibarra, Marcio Concepción-Zavaleta, Miguel Tipiani Mallma, Héctor Delgado Nicolás, Hilder Herrera-Silvestre, Laurie Marcilla-Truyenque, Joseph Arzapalo-Benavides, Luis Concepción-Urteaga

**Affiliations:** 1 Facultad de Medicina, Universidad Nacional Mayor de San Marcos, Lima, Perú.; 2 Hospital Nacional Edgardo Rebagliati Martins, Lima, Perú.; 3 Universidad Científica del Sur, Lima, Perú.; 4 Facultad de Medicina, Universidad Nacional de Trujillo, Trujillo, Perú.

**Keywords:** Insuficiencia adrenal, Enfermedad de Addison, Cryptococcus

## Abstract

Las micosis sistémicas, como la criptococosis, afectan principalmente los pulmones y el sistema nervioso central; sin embargo, la afectación de otros órganos, como las glándulas suprarrenales, es poco frecuente. Esto se ha descrito en algunos casos de insuficiencia suprarrenal primaria (ISP) de origen fúngico, los cuales se asocian con una alta mortalidad. Presentamos el caso de un hombre inmunocompetente de 65 años que presentó manifestaciones clínicas de insuficiencia suprarrenal. Las pruebas bioquímicas confirmaron ISP, y la tomografía abdominal reveló hiperplasia de ambas glándulas suprarrenales, predominantemente en el lado izquierdo. Se realizó una adrenalectomía izquierda y el examen patológico identificó granulomas y estructuras fúngicas compatibles con *Cryptococcus* spp. El paciente recibió terapia de reemplazo hormonal y tratamiento antifúngico, con una evolución favorable. La adrenalitis criptocócica debe considerarse en el diagnóstico diferencial de la ISP, especialmente en presencia de antecedentes epidemiológicos relevantes, y el tratamiento oportuno es clave para mejorar el pronóstico.

## INTRODUCCIÓN

La insuficiencia suprarrenal es un trastorno clínico caracterizado por la incapacidad de las glándulas suprarrenales para producir cantidades adecuadas de hormonas esteroides, principalmente cortisol y aldosterona. Se clasifica en insuficiencia suprarrenal primaria, secundaria y terciaria, según el origen de la disfunción. La insuficiencia suprarrenal primaria (ISP) es causada por la destrucción de las glándulas suprarrenales ^(^^1^.

Las etiologías de la insuficiencia suprarrenal primaria (ISP) incluyen causas autoinmunes, infecciosas, neoplásicas e infiltrativas. Las infecciones son una causa significativa de ISP, especialmente en regiones endémicas y en pacientes inmunocomprometidos ^1^. Históricamente, la tuberculosis ha sido la principal causa infecciosa de ISP ^2^; sin embargo, en la era moderna, la prevalencia de otras infecciones, como la adrenalitis criptocócica, ha aumentado debido a la expansión de la pandemia de VIH/SIDA y otros estados de inmunosupresión ^3^.

*Cryptococcus* spp. es un hongo encapsulado que causa criptococosis, una infección sistémica que afecta predominantemente a individuos con inmunodeficiencias graves. La infección se adquiere por la inhalación de esporas presentes en el ambiente, lo que permite su diseminación hematógena a diversos órganos, incluido el sistema nervioso central y las glándulas suprarrenales ^4^. Afecta principalmente a personas inmunocomprometidas, aunque también puede presentarse en huéspedes inmunocompetentes ^5^.

Las manifestaciones clínicas de la adrenalitis criptocócica pueden ser difíciles de distinguir de otras causas de insuficiencia suprarrenal. Los síntomas comunes incluyen fatiga, anorexia, pérdida de peso, hipotensión, hiperpigmentación cutánea, hipoglucemia, hiponatremia e hipercalcemia ^6^. Sin embargo, en la adrenalitis criptocócica, estos síntomas pueden ir acompañados de manifestaciones neurológicas o respiratorias debido a la diseminación sistémica de la infección, lo que complica aún más el diagnóstico ^4^.

El diagnóstico de la adrenalitis criptocócica requiere un alto índice de sospecha clínica. Los estudios de imagen, como la tomografía computarizada (TC) o la resonancia magnética de las glándulas suprarrenales, pueden revelar un agrandamiento suprarrenal bilateral ^7^. La confirmación diagnóstica se obtiene generalmente mediante cultivos fúngicos, prueba de antígeno criptocócica en suero o líquido cefalorraquídeo ^4^ y, en algunos casos, biopsia suprarrenal ^6^.

El tratamiento de elección para la criptococosis sistémica con afectación suprarrenal incluye terapia antifúngica con agentes como anfotericina B liposomal y flucitosina ^8^, esta última no disponible en Perú, junto con terapia de reemplazo hormonal (gluco y mineralocorticoides). En países como el nuestro, existen recomendaciones de uso y experiencia exitosa con el tratamiento basado en anfotericina B desoxicolato y fluconazol ^9^^,^^10^.

El inicio temprano del tratamiento es fundamental para evitar la crisis suprarrenal. La respuesta al tratamiento depende de la gravedad de la infección y del estado inmunológico del paciente, por lo que el monitoreo cercano es esencial ^11^. El objetivo de este reporte es describir el caso de un paciente masculino inmunocompetente de edad avanzada que desarrolló insuficiencia suprarrenal primaria causada por *Cryptococcus* spp.

## REPORTE DE CASO

Paciente peruano de 65 años, veterinario con más de 25 años de experiencia en el manejo de ganado. Nació en la ciudad de Huamanga, Ayacucho, Perú, pero ha residido en Lima, Perú, por más de 25 años. Fue diagnosticado con diabetes mellitus tipo 2 (DM2) hace 11 años y tratado con metformina 850 mg. Sus antecedentes médicos también incluyen un evento cerebrovascular isquémico hace 4 años, sin secuelas.

En diciembre de 2023, fue admitido en el Hospital Nacional Edgardo Rebagliati Martins, un hospital de referencia nacional, sus signos vitales fueron los siguientes: presión arterial de 80/60 mm Hg, frecuencia cardíaca de 97 latidos por minuto, afebril y sin signos meníngeos. Se encontraba desorientado y somnoliento, con hiperpigmentación de la piel y mucosas (Figura 1), sin masas abdominales palpables. Los análisis bioquímicos revelaron un nivel de sodio de 113 mmol/L y potasio de 5,2 mmol/L. Ante la sospecha de una crisis suprarrenal, se solicitó la medición de cortisol sérico y se inició hidratación junto con hidrocortisona 100 mg IV cada 8 horas. Se diagnosticó ISP y diabetes controlada con los siguientes resultados: Cortisol basal: 5,77 ug/dL (R.V: 3,7-19,4); ACTH plasmático: 197 pg/mL (R.V: 0-46); DHEA-S: 18,20 ug/dL (R.V.: 33,6-78,9); aldosterona plasmática: <10 pg/mL (R.V: 10-160); TSH: 1,12 uUI/mL (R.V: 0,4-4); T4 libre: 1,6 ng/dL (R.V: 0,8-1,9); Anticuerpos antitiroideos: (-); HbA1C: 6,1%; y se inició terapia con prednisona 7,5 mg/día y fludrocortisona 0,1 mg/día, logrando mejoría hemodinámica y bioquímica.

**Figura 1:** Hiperpigmentación en las flexuras de la palma y la mucosa gingival.

Como parte del estudio etiológico, se realizaron pruebas de esputo y lavado gástrico para bacilos ácido-alcohol resistentes, pancultivos y prueba de tuberculina, todas con resultados negativos; se realizó la prueba de criptolatex en suero, con resultado positivo. La serología TORCH (toxoplasmosis, rubéola, citomegalovirus y virus del herpes simple), así como la serología para hepatitis B y C, HTLV 1-2 y VIH, fueron negativas. Los anticuerpos antinucleares, antiperoxidasa tiroidea y antitiroglobulina también resultaron negativos. La radiografía de tórax fue normal, y la tomografía computarizada (TC) de glándulas suprarrenales mostró un agrandamiento bilateral, con un grosor de 11 mm en la glándula suprarrenal derecha y 14 mm en la izquierda, ambas con apariencia nodular hipodensa y valores de atenuación sin contraste de 45 HU y 25 HU para la suprarrenal derecha e izquierda, respectivamente (Figura 2). En una reunión multidisciplinaria, se recomendó adrenalectomía izquierda. El estudio anatomopatológico reveló inflamación granulomatosa con necrosis hemorrágica y estructuras fúngicas redondeadas compatibles con *Cryptococcus* spp. (Figuras 3 y 4). Se confirmó el diagnóstico de adrenalitis criptocócica causada por *Cryptococcus* spp.

**Figura 2:** Tomografía abdominal en corte transversal que muestra hiperplasia suprarrenal nodular bilateral con predominio en el lado izquierdo (flechas azules).

**Figura 3:** En la imagen macroscópica, la glándula suprarrenal izquierda presenta áreas de color pardo-amarillento con un centro necrótico y consistencia friable (a). En la imagen microscópica, se observa un extenso proceso inflamatorio granulomatoso crónico con necrosis, hemorragia y células gigantes multinucleadas que rodean estructuras redondeadas y ovaladas translúcidas (H&E, 40x) (b).

**Figura 4:** Imagen microscópica con una magnificación de 40x, las estructuras fúngicas redondeadas fueron teñidas mediante técnicas histoquímicas con tinción PAS (a) y tinción de Grocott (b), respectivamente.

Debido a la persistencia de cefalea holocraneal, predominantemente en la región occipital, se realizó una punción lumbar. Se obtuvo una presión de apertura de 15 cm de agua, la tinción con tinta china fue negativa, la aglutinación con látex para *Cryptococcus* resultó positiva (prueba cuantitativa 1:256) y el cultivo de LCR fue negativo. Se concluyó que el paciente presentaba compromiso del sistema nervioso central (SNC). El diagnóstico final fue criptococosis sistémica con afectación del SNC y de las glándulas suprarrenales. El paciente recibió tratamiento con fluconazol 400 mg/día y anfotericina B desoxicolato 1 mg/kg/día por vía intravenosa (fase intensiva) durante 2 semanas, seguido de 4 semanas adicionales de anfotericina B. El paciente mostró una evolución favorable y fue dado de alta con diagnóstico de insuficiencia suprarrenal primaria (ISP), con tratamiento ambulatorio de fluconazol oral por 6 meses (fase de consolidación), prednisona 5 mg una vez al día y fludrocortisona 0,1 mg una vez al día, los cuales continúa tomando. No se identificaron signos de toxicidad renal, hepática y cardiovascular asociados al uso de anfotericina B y fluconazol.

## DISCUSIÓN

La adrenalitis autoinmune es la causa principal de insuficiencia suprarrenal primaria (ISP), presentándose principalmente entre los 30 y 50 años, con una predilección por el sexo femenino ^12^^,^^13^. El caso reportado involucró a un paciente masculino de 65 años, lo que nos llevó a considerar otras causas de ISP. La TC suprarrenal, que mostró un agrandamiento bilateral de las glándulas suprarrenales, junto con los antecedentes del paciente como veterinario que trabajaba con ganado, nos llevó a realizar una biopsia suprarrenal para identificar la etiología.

La criptococosis es una enfermedad infecciosa causada por dos agentes: *Cryptococcus neoformans* y *Cryptococcus gattii*, que puede afectar los pulmones, las meninges, la piel, el sistema nervioso central y también las glándulas suprarrenales ^14^. Tradicionalmente, se considera una infección oportunista en pacientes inmunocomprometidos ^14^. Sin embargo, en los últimos años se ha observado un aumento en la incidencia de esta enfermedad en individuos inmunocompetentes y en aquellos con inmunodeficiencia leve a moderada. En estos casos, la infección puede ser asintomática y resolverse completamente sin tratamiento antifúngico, aunque, en raras ocasiones, puede progresar a una enfermedad grave con diseminación y desarrollo de meningitis criptococósica, como se observó en nuestro caso ^14^^,^^15^.

Las manifestaciones clínicas de la infección por *Cryptococcus* spp. son inespecíficas y pueden variar desde un cuadro insidioso hasta una presentación grave. La infección del sistema nervioso central puede manifestarse con cefalea, náuseas y vómitos, malestar general, alteración del estado mental, fiebre y convulsiones ^16^. Una presentación atípica y muy rara de la infección por *Cryptococcus spp* es la, que ocurre cuando el 90% o más de la corteza suprarrenal ha sido destruida ^17^, alcanzando las glándulas suprarrenales por vía hematógena debido a su extensa vascularización ^18^^,^^19^.

Se postula que *Cryptococcus spp* puede afectar las glándulas suprarrenales de dos maneras. La primera es de forma directa, donde *Cryptococcus neoformans* coloniza y prolifera en el tejido suprarrenal. El daño es causado por la invasión fúngica y la respuesta inflamatoria resultante. La cápsula fúngica, compuesta principalmente de polisacáridos como el glucuronoxilomanano, facilita la adherencia a las células adrenocorticales y la resistencia a la fagocitosis. Esta invasión conduce a una inflamación crónica y a la formación de granulomas en el tejido suprarrenal, causando adrenalitis. La inflamación local provoca necrosis del tejido adrenocortical, lo que lleva a insuficiencia suprarrenal ^18^^,^^20^. La segunda es de manera indirecta, donde la presencia de un agente infeccioso en otra parte del cuerpo activa el eje hipotálamo-hipófisis-suprarrenal. Esta activación prolongada conduce a hipercortisolismo, lo que podría afectar la función inmunológica, especialmente la inmunidad mediada por células T ^18^.

No existe un signo patognomónico para la adrenalitis criptocócica. La presentación clínica corresponde a la de una ISP y/o una crisis suprarrenal. En comparación con los pocos casos publicados, las principales manifestaciones clínicas reportadas incluyen fatiga, náuseas y vómitos, anorexia, dolor abdominal, deposiciones diarreicas, hipotensión, hiponatremia e hipopotasemia. Es importante destacar que no se han documentado casos de adrenalitis criptocócica aislada, sino que siempre ha sido parte de una diseminación desde un foco primario, generalmente meníngeo y/o pulmonar ^(^^21^^,^^22^^,^^23^.

Pocos estudios prospectivos ofrecen pautas específicas para el tratamiento de la criptococosis en pacientes sin VIH y sin trasplante, por lo que se siguen las mismas guías que para los pacientes con VIH. Aunque las guías de manejo actuales recomiendan el uso de agentes como anfotericina B liposomal y flucitosina como terapia de primera línea ^(^^8^, el uso de anfotericina B combinada con fluconazol, seguida de una fase de consolidación con fluconazol durante 6 a 12 meses, ha demostrado buenos resultados en la práctica clínica. Esto se evidenció en la evolución favorable de nuestro paciente con el tratamiento antifúngico instaurado ^24^^,^^25^.

El pronóstico de la función adrenal en pacientes con adrenalitis criptocócica que reciben tratamiento antifúngico varía y depende del grado de daño adrenal y la respuesta al tratamiento. Si bien, el uso de anfotericina B y fluconazol puede controlar la infección, el daño adrenal suele ser irreversible, lo que resulta en insuficiencia adrenal permanente y la necesidad de terapia de reemplazo hormonal a largo plazo ^18^^,^^20^.

En conclusión, la adrenalitis criptocócica es una afección muy rara, inespecífica y de difícil diagnóstico en pacientes sin inmunosupresión detectable. Sin embargo, es importante considerarla en el diagnóstico diferencial de la ISP. En el presente caso, el inicio oportuno del tratamiento antifúngico permitió una evolución clínica favorable para el paciente.
